# Supplementary material for: Systematic review and meta-analysis of artificial intelligence in classifying HER2 status in breast cancer immunohistochemistry
Source: NPJ Digit Med. 2025 Mar 6;8:144. doi: 10.1038/s41746-025-01483-8 (PMC11885561; doi:10.1038/s41746-025-01483-8)
Supplement: Supplementary file 1 — Supplementary Information [file 41746_2025_1483_MOESM1_ESM.pdf]

Supplementary Material Legends

- Supplementary Table 1: **PRISMA-DTA checklist.**
- Supplementary Table 2: **Description of QUADAS-AI risk of bias and applicability concerns questionnaire.**
- Supplementary Figure 1: **Forest plots of paired sensitivity and specificity for HER2 scores from 25 contingency tables.**
- Supplementary Table 3: **Analysis of threshold effect with Spearman correlation test.**
- Supplementary Table 4: **Subgroup analysis and meta-regression of AI in distinguishing HER2 scores 1+/2+/3+ *vs.* 0.**
- Supplementary Figure 2: **Analysis of publication bias with Deek's funnel plot.**
- Supplementary Table 5: **"Leave-one-out" sensitivity analysis of the 1+/2+/3+ *vs.* 0 meta-analysis.**
- Supplementary Figure 3: **Risk of bias and applicability concerns of the included studies using the adapted QUADAS-AI tool.**

Section/topic	#	PRISMA-DTA Checklist Item	Reported on page #
**TITLE / ABSTRACT**			
Title	1	Identify the report as a systematic review (+/- meta-analysis) of diagnostic test accuracy (DTA) studies.	1
Abstract	2	Abstract: See PRISMA-DTA for abstracts.	1
**INTRODUCTION**			
Rationale	3	Describe the rationale for the review in the context of what is already known.	1, 2
Clinical role of index test	D1	State the scientific and clinical background, including the intended use and clinical role of the index test, and if applicable, the rationale for minimally acceptable test accuracy (or minimum difference in accuracy for comparative design).	2
Objectives	4	Provide an explicit statement of question(s) being addressed in terms of participants, index test(s), and target condition(s).	2
**METHODS**			
Protocol and registration	5	Indicate if a review protocol exists, if and where it can be accessed (e.g., Web address), and, if available, provide registration information including registration number.	8
Eligibility criteria	6	Specify study characteristics (participants, setting, index test(s), reference standard(s), target condition(s), and study design) and report characteristics (e.g., years considered, language, publication status) used as criteria for eligibility, giving rationale.	8
Information sources	7	Describe all information sources (e.g., databases with dates of coverage, contact with study authors to identify additional studies) in the search and date last searched.	8
Search	8	Present full search strategies for all electronic databases and other sources searched, including any limits used, such that they could be repeated.	8
Study selection	9	State the process for selecting studies (i.e., screening, eligibility, included in systematic review, and, if applicable, included in the meta-analysis).	8
Data collection process	10	Describe method of data extraction from reports (e.g., piloted forms, independently, in duplicate) and any processes for obtaining and confirming data from investigators.	8
Definitions for data extraction	11	Provide definitions used in data extraction and classifications of target condition(s), index test(s), reference standard(s) and other characteristics (e.g. study design, clinical setting).	8
Risk of bias and applicability	12	Describe methods used for assessing risk of bias in individual studies and concerns regarding the applicability to the review question.	8
Diagnostic accuracy measures	13	State the principal diagnostic accuracy measure(s) reported (e.g. sensitivity, specificity) and state the unit of assessment (e.g. per-patient, per-lesion).	8
Synthesis of results	14	Describe methods of handling data, combining results of studies and describing variability between studies. This could include but is not limited to: a) handling of multiple definitions of target condition. b) handling of multiple thresholds of test positivity, c) handling multiple index test readers, d) handling of indeterminate test results, e) grouping and comparing tests, f) handling of different reference standards	8
Meta-analysis	D2	Report the statistical methods used for meta-analyses, if performed.	8
Additional analyses	16	Describe methods of additional analyses (e.g., sensitivity or subgroup analyses, meta-regression), if done, indicating which were pre-specified.	8
**RESULTS**			
Study selection	17	Provide numbers of studies screened, assessed for eligibility, included in the review (and included in meta-analysis, if applicable) with reasons for exclusions at each stage, ideally with a flow diagram.	2
Study characteristics	18	For each included study provide citations and present key characteristics including: a) participant characteristics (presentation, prior testing), b) clinical setting, c) study design, d) target condition definition, e) index test, f) reference standard, g) sample size, h) funding sources	2, 3, 4
Risk of bias and applicability	19	Present evaluation of risk of bias and concerns regarding applicability for each study.	5
Results of individual studies	20	For each analysis in each study (e.g. unique combination of index test, reference standard, and positivity threshold) report 2x2 data (TP, FP, FN, TN) with estimates of diagnostic accuracy and confidence intervals, ideally with a forest or receiver operator characteristic (ROC) plot.	5
Synthesis of results	21	Describe test accuracy, including variability; if meta-analysis was done, include results and confidence intervals.	4
Additional analysis	23	Give results of additional analyses, if done (e.g., sensitivity or subgroup analyses, meta-regression; analysis of index test: failure rates, proportion of inconclusive results, adverse events).	4, 5
**DISCUSSION**			
Summary of evidence	24	Summarize the main findings including the strength of evidence.	5, 6
Limitations	25	Discuss limitations from included studies (e.g. risk of bias and concerns regarding applicability) and from the review process (e.g. incomplete retrieval of identified research).	7
Conclusions	26	Provide a general interpretation of the results in the context of other evidence. Discuss implications for future research and clinical practice (e.g. the intended use and clinical role of the index test).	7
**FUNDING**			
Funding	27	For the systematic review, describe the sources of funding and other support and the role of the funders.	N/A

Supplementary Table 1: **PRISMA-DTA checklist.**

Domain	Signalling question
**Patient selection**	**Risk of bias**<br>• Is the source, size, and quality of the input data clearly defined, along with patient eligibility criteria?<br>• Was the data obtained from non-open-source datasets?<br>• Is there a clear rationale and distribution provided for training, validation, and test sets?<br>• Was image pre-processing performed?<br>• Is the scanner model used for imaging acquisition specified?<br>**Applicability concerns**<br>• Are there concerns that the included patients and setting do not match the review question?
**Index test**	**Risk of bias**<br>• Was external validation conducted?<br>**Applicability concerns**<br>• Are there concerns that the index test, its conduct, or interpretation differ from the review question?
**Reference standard**	**Risk of bias**<br>• Is the reference standard likely to correctly identify the target condition?<br>**Applicability concerns**<br>• Are there concerns that the target condition as defined by the reference standard does not match the question?
**Flow and timing**	**Risk of bias**<br>• Was the time between conducting the index test and the reference standard appropriate?

Supplementary Table 2: **Description of QUADAS-AI risk of bias and applicability concerns questionnaire**

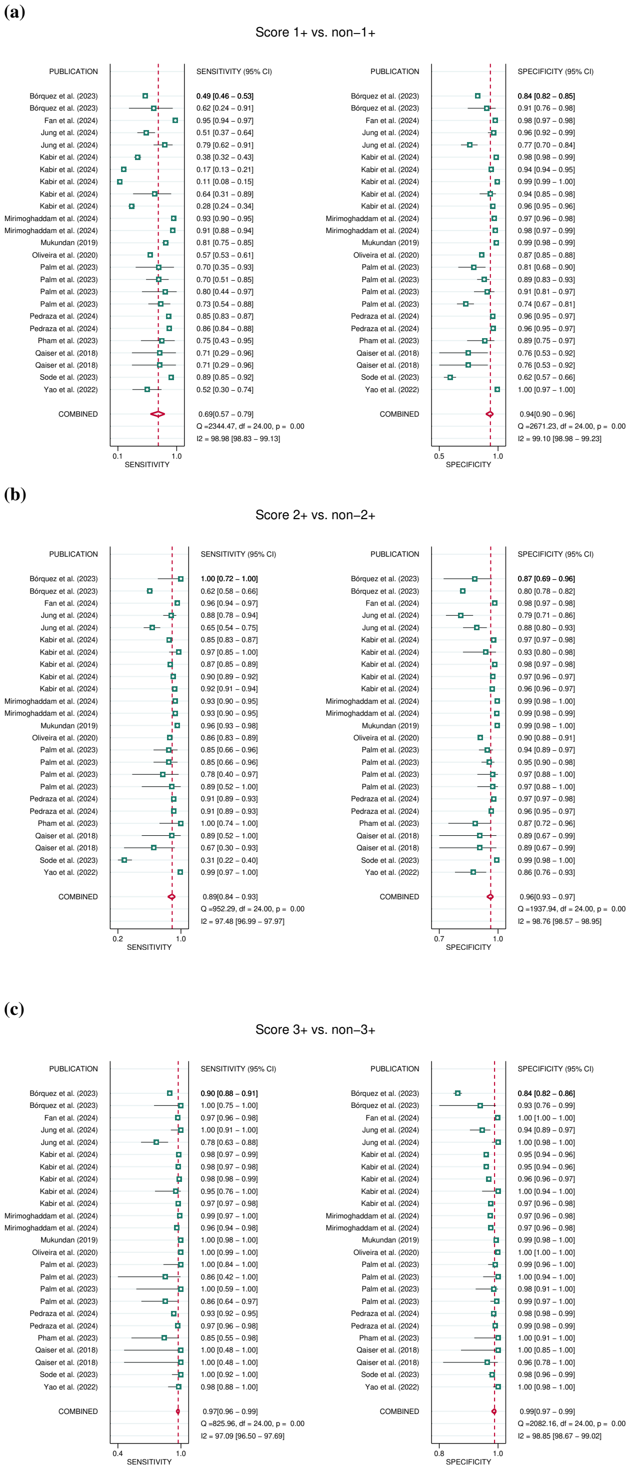

Supplementary Figure 1: **Forest plots of paired sensitivity and specificity for HER2 scores from 25 contingency tables.** **a** score 1+ *vs.* non-1+. **b** score 2+ *vs.* non-2+. **c** score 3+ *vs.* non-3+. Sensitivity and specificity increased with higher HER2 scores, demonstrating near-perfect performance at score 3+. The combined performances were calculated using the bivariate random-effects model. Heterogeneity was assessed using the Higgins inconsistency index statistic (I^2), showing high levels (≥75%) across all HER2 scores. CI, confidence interval.

HER2 cut-off	Spearman correlation coefficient	*p*-value
1+/2+/3+ *vs.* 0	0.050	0.811
1+	0.087	0.679
2+	-0.068	0.746
3+	0.107	0.610

Supplementary Table 3: **Analysis of threshold effect with Spearman correlation test**. Coefficients were computed between sensitivity and specificity using logit transformations. *p*-values indicate the absence of a threshold effect across the different HER2 cut-offs. HER2, Human Epidermal Growth Factor Receptor 2.

Covariate	Subgroup	Sensitivity [95% CI]	*p*-value	Specificity [95% CI]	*p*-value	N
Deep learning	No	0.94 [0.93 - 0.95]	$p < 0.001$	0.81 [0.67 - 0.90]	$p = 0.959$	6
	Yes	0.98 [0.97 - 0.99]		0.81 [0.71 - 0.89]		19
Commercially available algorithm	No	0.98 [0.97 - 0.99]	$p = 0.001$	0.83 [0.72 - 0.90]	$p = 0.121$	20
	Yes	0.93 [0.90 - 0.95]		0.73 [0.64 - 0.81]		5
External validation	No	0.98 [0.96 - 0.99]	$p = 0.006$	0.82 [0.67 - 0.91]	$p = 0.989$	15
	Yes	0.96 [0.95 - 0.97]		0.82 [0.74 - 0.88]		10
Sample size	≤761	0.98 [0.95 - 0.99]	$p = 0.897$	0.70 [0.55 - 0.82]	$p = 0.018$	13
	>761	0.97 [0.96 - 0.98]		0.88 [0.81 - 0.93]		12
Data unit	WSIs/cases	0.98 [0.96 - 0.99]	$p = 0.370$	0.70 [0.53 - 0.83]	$p = 0.048$	11
	Patches	0.97 [0.95 - 0.98]		0.87 [0.79 - 0.92]		14
Transfer learning	No	0.97 [0.95 - 0.99]	$p = 0.872$	0.76 [0.61 - 0.87]	$p = 0.229$	11
	Yes	0.98 [0.96 - 0.99]		0.85 [0.75 - 0.92]		14
Autonomy	Assisted	0.97 [0.92 - 0.99]	$p = 0.607$	0.77 [0.53 - 0.91]	$p = 0.614$	5
	Automated	0.98 [0.96 - 0.98]		0.83 [0.73 - 0.89]		20
Type of internal validation	Random split sample	0.98 [0.96 - 0.99]	$p = 0.492$	0.83 [0.66 - 0.93]	$p = 0.907$	8
	k-fold cross validation	0.98 [0.96 - 0.99]		0.82 [0.68 - 0.91]		12
Dataset	Own	0.98 [0.96 - 0.99]	$p = 0.619$	0.82 [0.72 - 0.90]	$p = 0.833$	9
	HER2SC	0.97 [0.96 - 0.98]		0.81 [0.68 - 0.89]		16

Supplementary Table 4: **Subgroup analysis and meta-regression of AI in distinguishing HER2 scores 1+/2+/3+ *vs.* 0.** *p*-values were obtained from the likelihood ratio test comparing models with and without the covariates using mixed-effect logistic regression. "N" represents the number of contingency tables utilised in each subgroup. CI, confidence interval; HER2SC, HER2 Scoring Contest; WSI, whole slide image

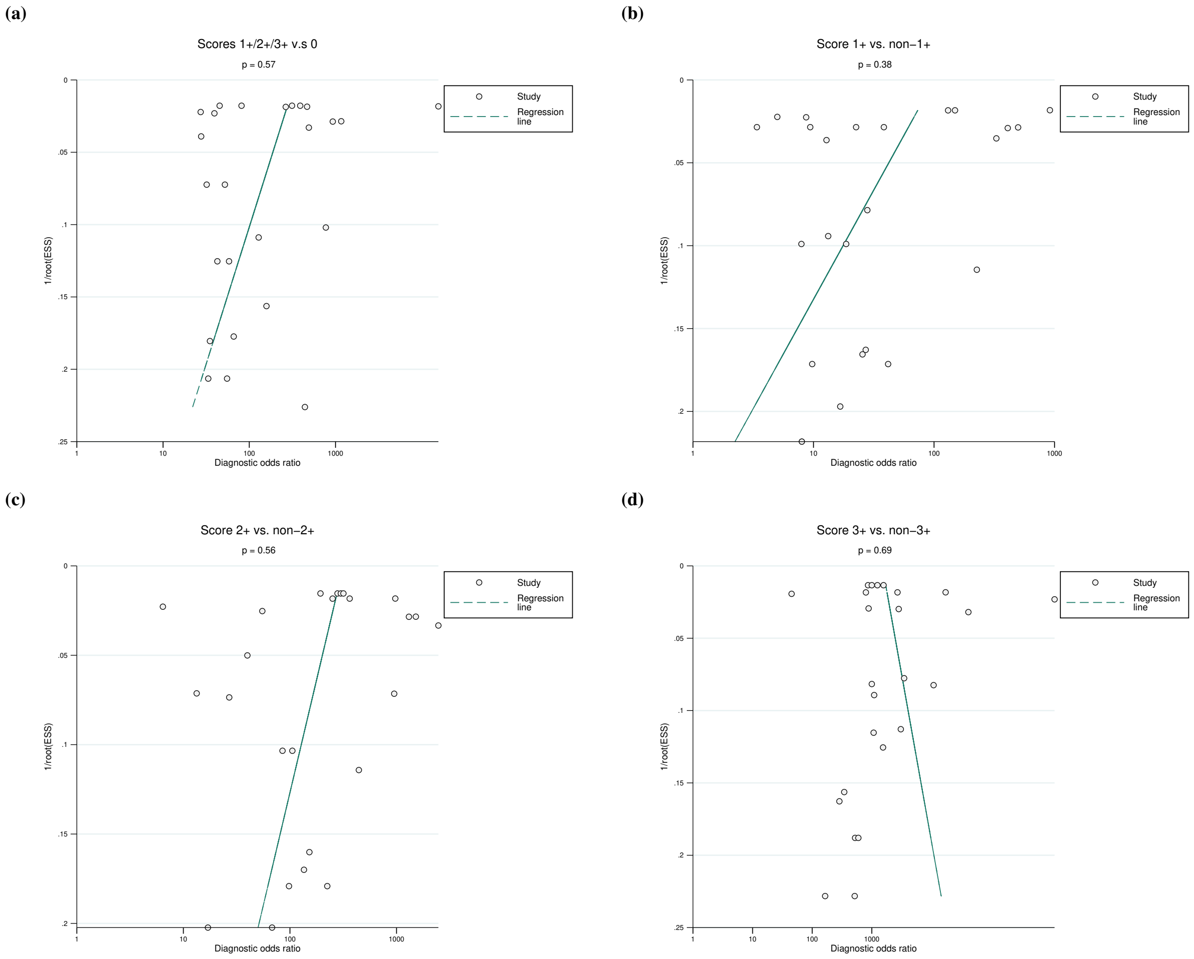

Supplementary Figure 2: **Analysis of publication bias with Deek's funnel plot.** **a** scores 1+/2+/3+ *vs.* 0. **b** score 1+ *vs.* non-1+. **c** score 2+ *vs.* non-2+. **d** score 3+ *vs.* non-3+. The studies demonstrated reasonable symmetry around the regression lines across all thresholds, with a non-significant effect, suggesting a low likelihood of publication bias. The asymmetry test was performed using a regression of the diagnostic log odds ratio, weighted by the $1/\sqrt{ESS}$. A $p < 0.10$ for the slope coefficient indicates significant asymmetry and high likelihood of publication bias. ESS, effective sample size.

Excluded study	Sensitivity [95% CI]	Specificity [95% CI]	AUC [95% CI]
Bórquez et al. (2023)	0.97 [0.96 - 0.98]	0.85 [0.79 - 0.90]	0.98 [0.96 - 0.99]
Fan et al. (2024)	0.97 [0.96 - 0.98]	0.80 [0.71 - 0.87]	0.98 [0.96 - 0.99]
Jung et al. (2024)	0.97 [0.96 - 0.98]	0.81 [0.72 - 0.88]	0.98 [0.96 - 0.99]
Kabir et al. (2024)	0.98 [0.97 - 0.99]	0.80 [0.69 - 0.88]	0.98 [0.96 - 0.99]
Mirimoghaddam et al. (2024)	0.97 [0.96 - 0.98]	0.80 [0.70 - 0.87]	0.98 [0.96 - 0.99]
Mukundan (2019)	0.98 [0.96 - 0.98]	0.80 [0.71 - 0.87]	0.98 [0.96 - 0.99]
Oliveira et al. (2020)	0.98 [0.96 - 0.98]	0.82 [0.73 - 0.88]	0.98 [0.96 - 0.99]
Palm et al. (2023)	0.98 [0.97 - 0.99]	0.82 [0.72 - 0.89]	0.98 [0.97 - 0.99]
Pedraza et al. (2024)	0.98 [0.96 - 0.98]	0.80 [0.70 - 0.87]	0.98 [0.96 - 0.99]
Pham et al. (2023)	0.97 [0.96 - 0.98]	0.82 [0.73 - 0.88]	0.98 [0.96 - 0.99]
Qaiser et al. (2018)	0.97 [0.96 - 0.98]	0.83 [0.75 - 0.89]	0.98 [0.96 - 0.99]
Sode et al. (2023)	0.98 [0.96 - 0.98]	0.82 [0.73 - 0.88]	0.98 [0.96 - 0.99]
Yao et al. (2022)	0.97 [0.96 - 0.98]	0.82 [0.73 - 0.88]	0.98 [0.96 - 0.99]
**Combined**	**0.97 [0.96 - 0.98]**	**0.82 [0.73 - 0.88]**	**0.98 [0.96 - 0.99]**

Supplementary Table 5: **"Leave-one-out" sensitivity analysis of the 1+/2+/3+ *vs.* 0 meta-analysis**. Each row represents the performance when the corresponding study was excluded at a time from the overall meta-analysis. CI, confidence interval.

(a)

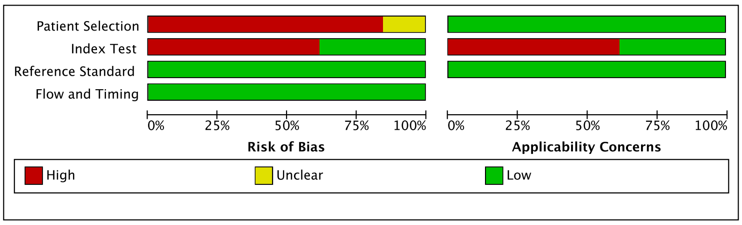

(b)

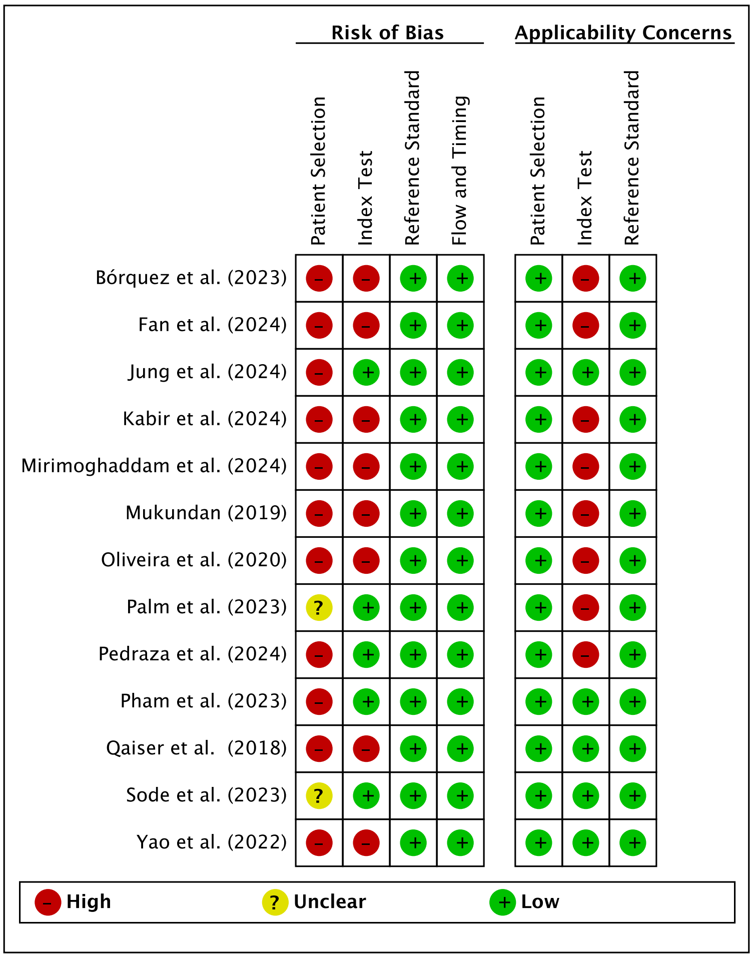

Supplementary Figure 3: **Risk of bias and applicability concerns of the included studies using the adapted QUADAS-AI tool.** **a** Risk of bias and applicability concerns presented as percentages of frequency across all included studies. **b** Summary of the authors' judgments on each risk of bias and applicability concern item across the thirteen included studies. Eleven studies were found to have a high risk of bias in the "patient selection" domain due to insufficient reporting of eligibility criteria, such as the method of tissue extraction (biopsy *vs.* resection), tumour invasiveness, and whether the tumours were primary or metastatic; additionally, two studies that employed commercially available algorithms failed to provide a clear rationale or a detailed breakdown of their training and validation datasets. The "index test" domain also exhibited a high risk of bias in eight studies due to the absence of external validation. Eight studies that assessed AI performance using patches raised high applicability concerns regarding their index tests.
